# Low-Cost and Device-Free Human Activity Recognition Based on Hierarchical Learning Model

**DOI:** 10.3390/s21072359

**Published:** 2021-03-28

**Authors:** Jing Chen, Xinyu Huang, Hao Jiang, Xiren Miao

**Affiliations:** College of Electrical Engineering and Automation, Fuzhou University, Fuzhou 350108, China; chenj@fzu.edu.cn (J.C.); 180120071@fzu.edu.cn (X.H.); mxr@fzu.edu.cn (X.M.)

**Keywords:** human activity recognition (HAR), coarse-to-fine hierarchical learning, gated recurrent unit (GRU)

## Abstract

Human activity recognition (HAR) has been a vital human–computer interaction service in smart homes. It is still a challenging task due to the diversity and similarity of human actions. In this paper, a novel hierarchical deep learning-based methodology equipped with low-cost sensors is proposed for high-accuracy device-free human activity recognition. ESP8266, as the sensing hardware, was utilized to deploy the WiFi sensor network and collect multi-dimensional received signal strength indicator (RSSI) records. The proposed learning model presents a coarse-to-fine hierarchical classification framework with two-level perception modules. In the coarse-level stage, twelve statistical features of time–frequency domains were extracted from the RSSI measurements filtered by a butterworth low-pass filter, and a support vector machine (SVM) model was employed to quickly recognize the basic human activities by classifying the signal statistical features. In the fine-level stage, the gated recurrent unit (GRU), a representative type of recurrent neural network (RNN), was applied to address issues of the confused recognition of similar activities. The GRU model can realize automatic multi-level feature extraction from the RSSI measurements and accurately discriminate the similar activities. The experimental results show that the proposed approach achieved recognition accuracies of 96.45% and 94.59% for six types of activities in two different environments and performed better compared the traditional pattern-based methods. The proposed hierarchical learning method provides a low-cost sensor-based HAR framework to enhance the recognition accuracy and modeling efficiency.

## 1. Introduction

Recently, the rapid development of the Internet of Things (IoT) technology and artificial intelligence has promoted the mutual “communication” between people and things and between things, greatly improving the lifestyle and quality of life of human beings. Human activity recognition (HAR), as a new human–computer interaction technology, has become one of the research hotspots in the field of IoT. The existing HAR technology can be divided into three types according to the implementation methods, namely, camera-based [1,2], sensor-based [3,4] and wireless signal-based [5]. The wireless signal-based recognition method has attracted more and more attention for its advantages of not being affected by indoor light, not requiring users to wear additional equipment and protecting privacy. With the widespread deployment of WiFi devices in indoor environments, the number of applications using WiFi wireless signals for human sensoring is also increasing. These applications include activity recognition [6,7], gesture recognition [8], occupancy detection [9], and human localization [10,11,12]. The basic principle behind these applications is that the presence and movement of the human body can change the way WiFi signals travel through the measurable receiver [13]. Intuitively, when a person appears in the sensing area of a WiFi transceiver pair, their body movement will affect the WiFi propagation path and thus lead to signal changes. By measuring and analyzing such signal changes at the receiving end, the realization of the human perception task is feasible. Currently, HAR based on WiFi usually includes two types of wireless signals, namely received signal strength indicator (RSSI) [14] and channel state information (CSI) [15]. RSSI is the measurement of radio signal power at the receiving terminal, and it is a kind of coarse-grained information belonging to the media access control (MAC) layer. Compared to CSI, it can be easily measured using most wireless devices because RSSI is supported by almost all wireless chips.

According to recognition techniques, existing activity recognition applications based on WiFi-RSSI can be divided into two types: traditional pattern-based applications and deep learning-based applications. The key issue of traditional pattern-based HAR is how to extract representative features from RSSI measurements and construct a model to effectively recognize human activities. Sigg et al. [14] designed an activity recognition system based on RSSI obtained from mobile phones, which combined four time domain features (mean, variance, maximum and difference between maximum and minimum) and the k-nearest neighbor (KNN) algorithm to realize activity recognition. The accuracy of this system is 51% when recognizing eleven gestures and 72% when recognizing four different gestures. However, a few statistical features provide only partial information and are susceptible to random noise, so the accuracy of identification is not satisfactory. In order to overcome this drawback, Wang et al. [16] obtained wavelet features from RSSI measurements to achieve device-free simultaneous wireless localization and activity recognition (DFLAR). Compared with time domain features, wavelet features could characterize the RSSI link measurements in both the time domain and frequency domain, thus providing further robust identification information and improving the performance of the DFLAR system. The experimental results showed that the accuracy of the location estimation and activity recognition can reach more than 90%. The above studies mainly focused on providing better solutions for RSSI-based activity representation, whilst paying less attention to activity classification algorithms. It should be noted that feature extraction and activity recognition are not mutually optimized. Therefore, Gu et al. [17] adopted the mean and variance of time domain for the characterization of RSSI measurements, and put forward a fusion algorithm based on a classification tree to identify the human activity. The fusion algorithm improved the recognition accuracy of similar activities, with an average recognition accuracy of 72.47%, and its performance was better than the other three famous classifiers (naive Bayes, Bagging and KNN) in terms of accuracy and complexity. Their work is illuminating, but its low accuracy is a major problem.

In contrast to the traditional pattern-based methods, deep learning-based methods can automatically extract or construct complex features through the intermediate network layers, and obtain a strong nonlinear fitting ability for input data and output tags. In recent years, it has been widely applied in the field of activity recognition. Wang et al. [6] used a three-layer deep learning network for feature extraction and realized DFLAR recognition simultaneously. The experimental results showed that the DFLAR system using deep learning features could achieve a precision of 85% or higher. Compared with the traditional manual feature extraction method, this method improves the recognition accuracy by 10%. Huang et al. [18] proposed WiDet, a human detection system based on WiFi-RSSI. This system used the deep learning method, namely the convolutional neural network (CNN), to automatically learn the features from the sensor sequence data, and combined the original RSSI measurements with the wavelet coefficients as the input of the neural network, which can distinguish the signal change caused by human movement from the random noise interference, and improve the detection accuracy of walking behavior to 95.5%. However, building a deep learning model requires a lot of computational cost, because the network parameters that need to be learned usually contain tens of thousands to millions.

In this paper, a novel low-cost coarse-to-fine hierarchical classification method based on an RSSI acquisition system with ESP8266 is proposed for HAR. The ESP8266 (manufactured by Espressif Systems, Shanghai, China), as the sensing hardware of HAR system, uses sniffer devices to actively measure RSSI values between multiple sensor nodes to ensure the adequacy of sensor information. A low-pass butterworth filter was introduced to filter out signal fluctuations and a small amount of abnormal data ensuring the availability of sensing information. The proposed hierarchical learning framework combines a pattern-based method with signal statistical features to detect the coarse-level activities, and a deep learning model for the secondary detection to accurately identify the similar activities. It is divided into two stages. At the coarse-level identification stage, twelve statistical features in the time domain and frequency domain are designed for the influence of human activity on wireless RSSI measurements. The differentiation of features between activities is enhanced by the fusion of multi-dimensional sensor signals in space. The support vector machine (SVM) is incorporated into the hierarchical recognition framework for the coarse-level classification of signal statistical features and activities. At the fine-level identification stage, to further distinguish similar human activities, an recurrent neural network (RNN) model with a gated recurrent unit (GRU) [19] blocks is utilized to learn the representative features and encode the temporal information for identifying similar activities. The GRU neural network can capture the complicated non-linear relationship between input and output data and realize the fine-level recognition of similar activities.

The main contributions of this paper are as follows:

1. A convenient RSSI acquisition system was developed based on NodeMCU with its prominent advantages of low-cost (a NodeMCU costs about USD 2.50.), stable and easy to deploy. The system utilizes the sniffer technique to obtain the RSSI measurements of multiple sensor links and performs signal cleaning through a low-pass butterworth filter, which ensures the adequacy and reliability of sensing information.
2. In contrast to the existing RSSI-based methods, a hierarchical classification framework combined the traditional pattern-based method and the deep learning-based method to realize high-accuracy activity recognition and simultaneously reduce the high training cost.
3. Twelve statistics in the time domain and frequency domain were designed to represent the influence of human activity on RSSI measurement, and an SVM classifier was constructed for coarse activity recognition, compared with other traditional pattern-based methods, achieving better coarse recognition performance.
4. To accurately recognize similar activities, the GRU model was leveraged to identify similar activities by extracting activity features from the contextual relationship between sensor signal frames, which can greatly enhance the recognition accuracy.

## 2. System Overview

**Figure 1:**
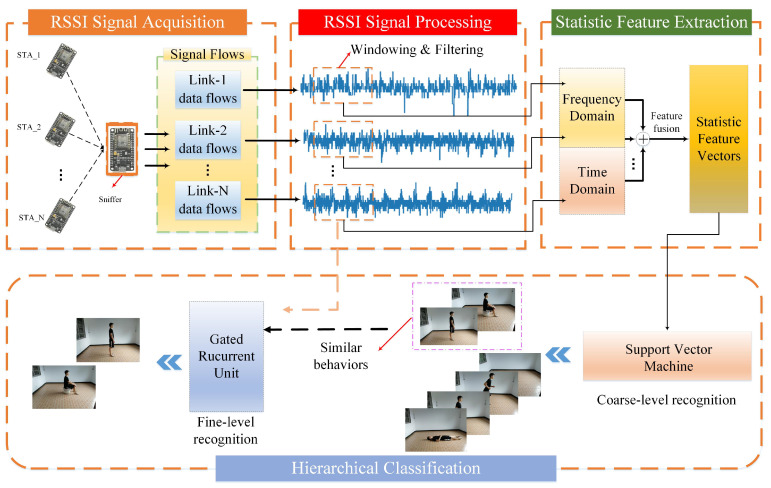
System framework of the hierarchical learning-based human activity recognition (HAR) method.

Aiming to produce low-cost, lightweight and high-precision human activity recognition, we proposed a novel hierarchical recognition framework based on WiFi-RSSI wireless sensing information using ESP8266 as the sensor hardware. Considering the degree of similarity between diverse human activities, the proposed hierarchical learning method was implemented through two stages of activity recognition. SVM was used to build a simple classifier for all activities, which achieved high accuracy recognition for most activities with large differences. This stage was defined as coarse-level recognition. Coarse-level classifiers have obvious mutual misrecognition phenomena for some similar activities. Furthermore, we use a GRU method to build a classifier only for such similar activities, and improved the activity recognition accuracy at the lowest model training cost. This stage was defined as fine-level recognition. As shown in Figure 1, our framework follows a modular structure consisting of five parts as follows:

### 2.1. RSSI Signal Acquisition

In order to improve the RSSI-based activity resolution capability, sensor nodes were deployed at different locations in the target environment, and multi-dimensional RSSI perception information was obtained to detect human activities by collecting RSSI measurements of multiple links between the detection device and each sensor node. According to the set sampling frequency, the acquisition system continuously received RSSI measurements to form sensing signal flow. In the data set collection stage, volunteers independently made different activities in the indoor environment with deployed sensors. The acquisition system collected the corresponding RSSI signals and established labels for the collected data set according to the types of activities.

### 2.2. Signal Preprocessing

After the data collection, the sliding window technology was used to cut the sensing signal flow, and the signal fragments of each measurement link were fused to form the signal samples of the activity. Then, a butterworth low-pass filter was utilized to filter out the noise of the signal to ensure the reliability of sensing information.

### 2.3. Statistical Feature Extraction

According to the pre-processed RSSI signal sequence, seven statistics in the time domain could be directly calculated, and five statistics in the frequency domain could be calculated after the fast Fourier transform (FFT) operation. The statistical features extracted from all RSSI measurement links were fused to form a feature vector describing human activities that was used as input for the activity in the coarse recognition model.

### 2.4. Activity Coarse Recognition

In the coarse-level recognition stage, the activity recognition was transformed into a classification problem of a statistical feature vector. We introduced the SVM algorithm to construct a low-complexity classification model for all activities, and took the statistical feature vector of the signal as the input of the model to recognize most activity categories with high accuracy. A few activities that were incorrectly recognized in the recognition result were identified as similar activities, which was realized in the secondary recognition by the subsequent fine model.

### 2.5. Activity Fine Recognition

In the fine-level recognition stage, according to the similar activities determined based on the coarse-level recognition results of the SVM model, the GRU recurrent network was introduced to directly construct the fine recognition model from the sensing signals of such activities. GRU can extract more effective features from the time dependence of RSSI measurements for similar activities. When a similar activity is identified at the coarse-level, the well-trained fine-level recognition model will be triggered to further recognize similar activities with high precision.

## 3. Signal Acquisition and Preprocessing

### 3.1. Signal Acquisition System

We developed an RSSI acquisition system based on a WiFi probe for human activity recognition. According to the IEEE802.11 protocol, a wireless terminal with WiFi enabled will regularly notify the existence of the network by broadcasting probe requests to the surrounding area through active scanning. A probe request frame is a subtype of the management frame that contains a terminal’s unique media access control (MAC) address. WiFi probes listen for the wireless frames of terminals with specific MAC addresses in promiscuous mode and obtain RSSI by parsing the the 802.11 frame.

**Figure 2:**
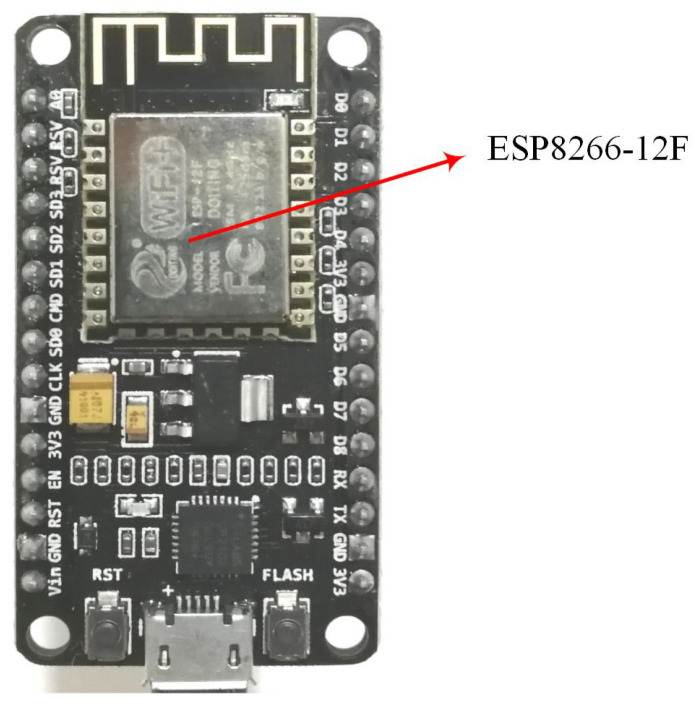
NodeMCU with the ESP8266-12F WiFi module.

The hardware of the RSSI acquisition system in this paper was based on the open source IoT platform called NodeMCU, as shown in Figure 2. Its core chip is Nodemcu-12F (manufactured by Espressif Systems, Shanghai, China), which is an in-built 32-bit micro-MCU with ultra-low power consumption and supports primary frequencies between 80 MHz and 160 MHz. The module integrates onboard WiFi antennas and supports the standard IEEE802.11 b/g/n protocol. The advantages of the hardware include its low cost, easy programming, stable performance, low power consumption and so on. NodeMCU, which itself acts as a sensor node, can be used as access point (AP) or as a station (STA).

**Figure 3:**
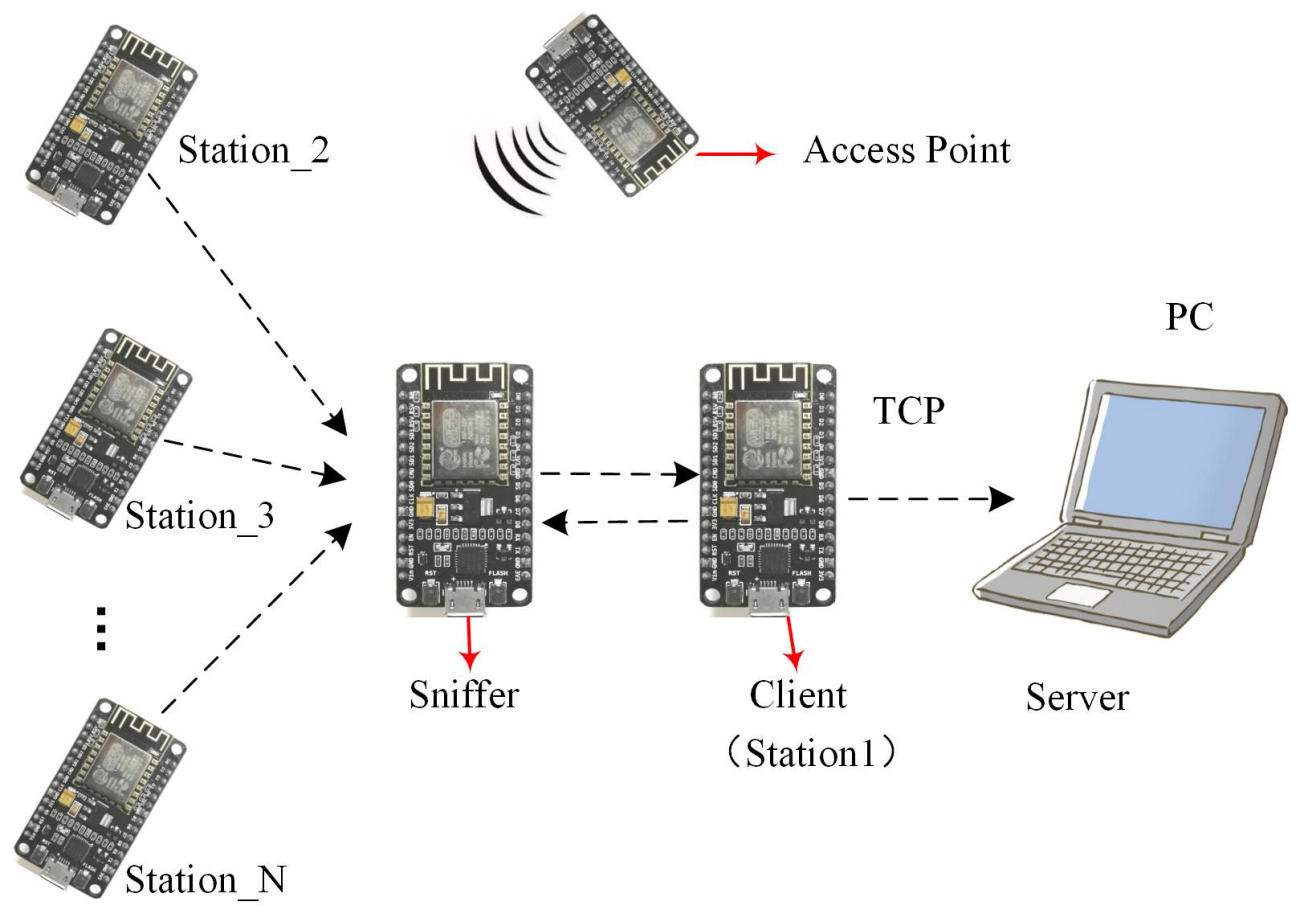
RSSI acquisition system architecture.

As shown in Figure 3, the acquisition system consists of four parts: the access point (AP), terminal group, a probe set and a server. The terminal group consists of a certain number of NodeMCU boards whose working mode is STA, and each NodeMCU is fixed in a different position in the experimental environment. A probe set is composed of two NodeMCU boards, which are communicated by serial port. One acts as a sniffer to detect the RSSI between itself and terminals at various locations, and the other acts as a client to transmit the detected RSSI data to the server based on the transmission control protocol (TCP).The server, which is a laptop, stores the information from the probe set and performs subsequent operations related to human activity recognition.

### 3.2. Signal Preprocessing

#### 3.2.1. Segmentation

Sliding window technology was introduced to cut the collected RSSI signal stream, and the signal after windowing was used for the subsequent preprocessing and statistical feature extraction module. The window size was set to *W*, and in order to maximize the number of training samples, we chose the step size of the sliding window to be 1. Therefore, for the RSSI signal stream with a length of *L*, the number of samples could be obtained to be L−W+1.

Assuming that there are a total of *N* available RSSI fragments after data segmenting, the RSSI vector *R* can be defined as

(1) $$R=\left[R_{1},...,R_{n},...,R_{N}\right]\;,\;\left(1\leq n\leq N\right)$$

where $R_{N}$ is the RSSI measurement matrix in the *n*th segment, which can be expressed as

(2) $$R_{n}={\left[r_{n}^{1},...,r_{n}^{j},...,r_{n}^{J}\right]}_{W\times J},\;\left(1\leq j\leq J\right)$$

where $r_{n}^{j}$ is the RSSI vector of the *j*th measurement link under the *n*th window, the dimension is W×1, and the total number of measurement links is *J*. The data are then labeled and provided to the GBDTclassifier based on supervised learning. The label vector was denoted as Label:

(3) $$Label=\left[L_{1},...,L_{n},...,L_{N}\right],\;\left(1\leq n\leq N\right)$$

where $L_{n}\in \left\{0,1,2,3,4,5,6\right\}$ is the label of the *n*th RSSI matrix, and 0, 1, 2, 3, 4, 5 and 6 represent lying, running, sitting, standing, walking, empty and sleeping, respectively.

#### 3.2.2. Signal Filtering

**Figure 4:**
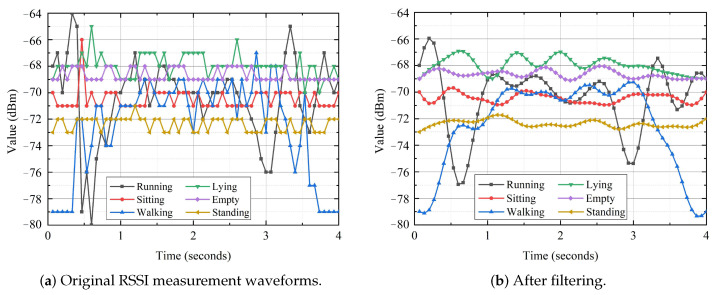
RSSI waveforms before and after filtering.

The premise of RSSI-based human activity sensoring is to obtain reliable RSSI measurements from WiFi devices. Raw RSSI measurements include random noises from various sources and abnormal measurements caused by the fluctuation of a WiFi device’s own power. These unexpected data are able to add false edges, and further influence the accuracy and robustness of activity identification. Furthermore, the frequency of human activity is in the low-frequency range, and many high-frequency noises contained in the RSSI signal need to be filtered out [20]. The role of signal preprocessing is to filter noises, remove outliers, and retain valuable data, which can be realized by using a low-pass butterworth filter. Taking the time series waveforms of RSSI measurements collected by the same volunteer under different activities as an example, the RSSI amplitude waveforms before and after denoising are shown in Figure 4. It can be easily observed that the denoised signals are smoother with a low noise level.

### 3.3. Statistical Feature Extraction

Statistical feature extraction refers to the calculation and analysis of the data by means of statistical methods. According to the properties of the sensing signal, the statistical features of the signal can usually be calculated in the time domain and frequency domain. Some applications implement the corresponding functionality using the statistics feature, such as heart disease prediction based on wireless body sensor network signals [21], and a through-wall human activity recognition system called WiHACS [22]. The intuition behind feature extraction is to transform the signal into a feature vector-depicting activity.

We can calculate the statistical characteristics based on the original time domain data and the data after the FFT operation. Seven statistical characteristic values were calculated for $r_{n}^{j}\left(1\leq j\leq J,1\leq n\leq N\right)$ in the time domain, namely the mean; standard deviation (SD); root mean square (RMS); maximum (Max); minimum (Min); range; and mean crossing (MC). At the same time, five statistical features of $r_{n}^{j}\left(1\leq j\leq J,1\leq n\leq N\right)$ after FFT were calculated, including the amplitude mean (AM); amplitude standard deviation (ASD); amplitude skewness (AS); amplitude kurtosis (AK); and shape mean (SM). Table 1 shows a list of some unusual frequency domain statistical calculation formulas, where x(i) is the *i* th RSSI measurement value in the window, C(i) is the *i* th frequency amplitude value of the window, and *W* is the size of the window.

Twelve statistical features were obtained for the RSSI sequence $r_{n}^{j}\left(1\leq j\leq J,1\leq n\leq N\right)$ of the *j*th link of the *n* th segment, and a eigenvector $f_{n}^{j}$ witha dimensiono *f* 1×12 can be obtained by splicing. Furthermore, we conducted eigenvector fusion for multiple sensor links in space. Therefore, for the *n* th window, the corresponding feature vector is:

(4) $$f_{n}={\left[f_{n}^{1},...,f_{n}^{j},...,f_{n}^{J}\right]}_{1\times 12J},\;\;\;\left(1\leq n\leq N\right)$$

Through the feature fusion of multiple sensor links, the *n* th RSSI measurement matrix is transformed into a feature vector $f_{n}$ with a dimension of 1×12J to describe human activity, and the human activity recognition is further transformed into a problem to classify the feature vectors.

## 4. Activity Recognition Model

### 4.1. SVM Recognition Model

In this paper, SVM was applied to construct the coarse-level recognition mode, and the activity recognition was realized by classifying the statistical feature vectors of signals. The labeled training data set can be expressed as $S_{1}=\left\{\left(f_{1},L_{1}\right),...,\left(f_{n},L_{n}\right),...,\left(f_{N},L_{N}\right)\right\}$, where *N* is the number of samples, and $f_{n}$ is the corresponding feature vector of the *n* window, and $L_{n}$ is the label of the corresponding segment. SVM is a machine learning algorithm proposed according to the principle of structural risk minimization [23]. It can learn better models without a large number of samples, and has good generalization performance and strong stability. The basic idea is to map the input space to a high-dimensional feature space through the nonlinear change defined by the inner product function, and to find the hyperplane that maximizes the margin between the training samples of different categories in this high-dimensional space, so that the samples are linearly separable in this space [24].

The generated classification model is represented as a boundary in the data mapping space, as shown below:

(5) $$f\left(x\right)=w^{T}\cdot \varphi \left(x\right)+b\;$$

where *w* represents the weight parameter, *b* represents the threshold value of the separated hyperplane, and φ(x) represents a nonlinear transformation function in a high-dimensional space.

Solving the optimal hyperplane is transformed into an optimization problem of structural risk objective function:

(6) $$\begin{array}{c} \min\;\;\;\;\;\;\;\;\;\;\frac{1}{2}{∥w∥}^{2}+C\sum_{i=1}^{N}\xi_{i}\\ s.t.\;\;\;\;\;\;\;\;\;\;\;y_{i}\left(w^{T}\cdot \varphi \left(x_{i}\right)+b\right)\geq 1-\xi_{i}\\ \;\;\;\;\;\;\;\;\;\;\;\;\;\;\;\;\;\;\;\;\;\;\xi_{i}\geq 0,\;\;\;\;i=1,2,\cdots ,N \end{array}$$

where $\xi_{i}$ is the slack factor, the purpose of which is to relax the restrictive conditions and allow a certain degree of misclassification; *C* is the penalty factor used to control misclassification; and *N* is the total number of samples.

A kernel function is introduced to solve the linearly inseparable problems. Let $K(x_{i},x_{j})$ be a kernel function, and let φ(x) be the associated feature mapping introduced by the kernel function. In this paper, a “polynomial” kernel function is selected:

(7) $$\left\{\begin{array}{c} K\left(x_{i},x_{j}\right)={\left(\left(x_{i}\cdot x_{j}\right)+1\right)}^{q} \end{array}\right.$$

where *q* is the polynomial degree of the kernel.

Generally, Lagrange duality is used to transform the optimization problem in Equation (6) into a simpler dual problem. The simplified dual problem is expressed here as

(8) $$\begin{array}{c} \min_{a}\;\;\;\;\;\;\;\;-\sum_{i=1}^{N}a_{i}+\frac{1}{2}\sum_{i=1}^{N}\sum_{j=1}^{N}a_{i}a_{j}y_{i}y_{j}K\left(x_{i},x_{j}\right)\\ \;\;\;s.t.\;\;\;\;\;\;\sum_{i,j=1}^{N}y_{i}a_{i}=0\;\;\;\;\;\;\;\;C\geq a_{i}\geq 0,i=1,2,\cdots ,N \end{array}$$

where $\alpha ={\left(\alpha_{1},\alpha_{2},\cdots ,\alpha_{N}\right)}^{T}$, and $\alpha_{i}$ is the Lagrange multiplier.

The decision function model obtained by solving the optimization problem of Equation (8) is as follows:

(9) $$\begin{array}{c} F_{1}\left(x\right)=\mathrm{sgn}\left\{\sum_{i=1}^{N}{\alpha_{i}}^{*}y_{i}K\left(x_{i},x_{j}\right)+b^{*}\right\}\\ \mathrm{where}\;\;\;b^{*}=y_{j}-\sum_{i=1}^{N}y_{i}\alpha_{i}^{*}K\left(x_{i},x_{j}\right) \end{array}$$

where, $\alpha_{i}^{*}$ and $b^{*}$ are the optimal values for $a_{i}$ and *b*, respectively; sgn(·) is a symbolic function, $j\in \left\{j|0<\alpha_{j}^{*}<C\right\}$. The classification labels of the test samples can be predicted by using the learned classifier given in Equation (9).

### 4.2. GRU Recognition Model

**Figure 5:**
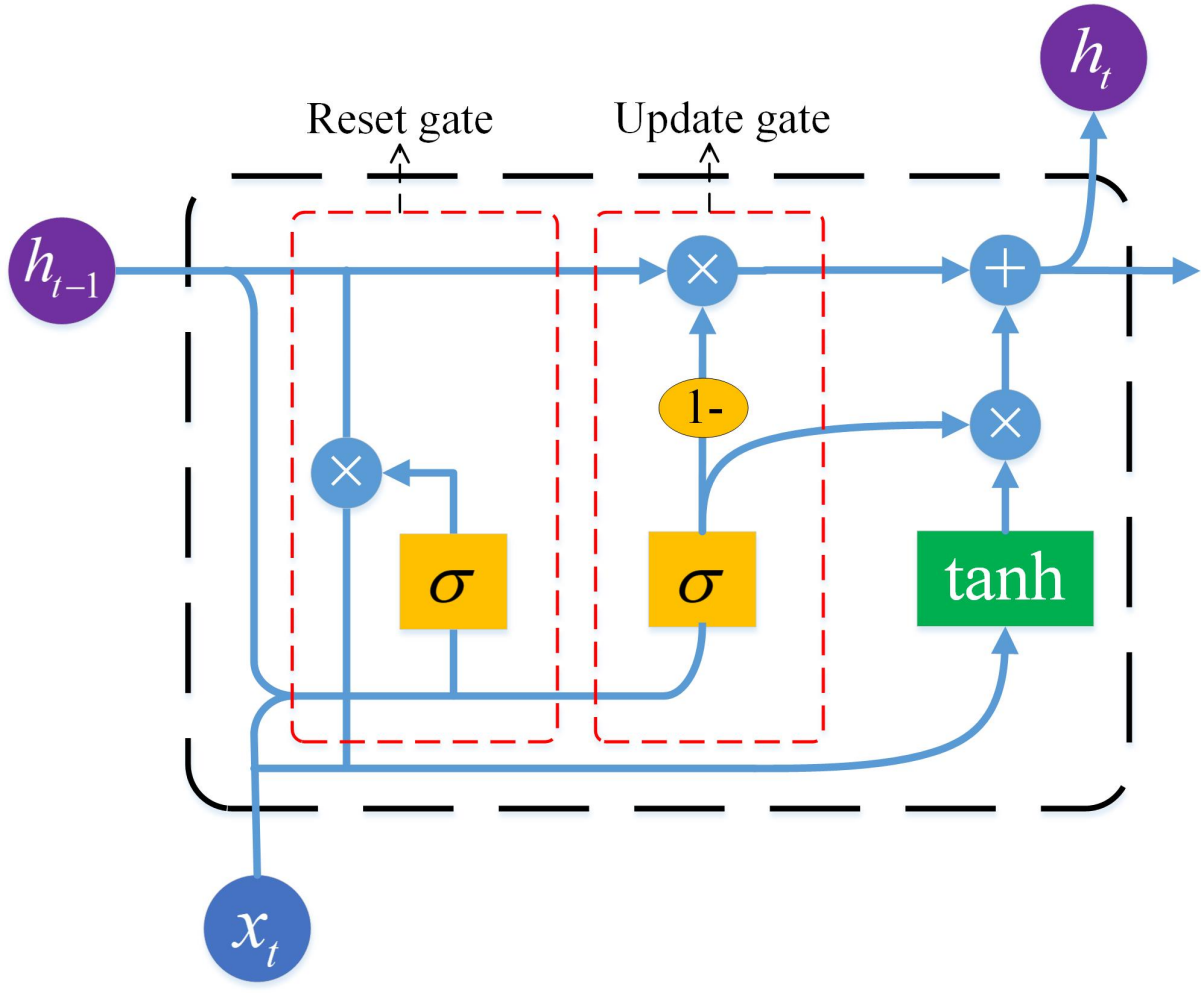
Structure of the gated recurrent unit (GRU) recurrent network.

Deep learning mostly adopts multi-layer neural network, which is composed of an input layer, multiple hidden layers and an output layer. Compared with the shallow network, the increase in the number of layers makes it possible to extract the essential features deeper in the data, which can obtain a better recognition effect. RNN is a representative neural network in deep learning. The nodes in its hidden layer are connected with each other, and the output of each node is not only related to the current input, but also depends on the output of the previous node. Therefore, it has a certain memory function, making it possible to explore the temporal relationship between discontinuous data [25]. However, it is worth noting that the vanishing gradient problem, caused by backpropagation, results in its limited ability to learn complex long sequences [26]. Long short-term memory (LSTM) [27] and GRU [19] neural networks, as variants of RNN, are proposed to solve the problem of the gradient disappearance of RNN. Compared with LSTM, GRU retains the function of LSTM while reducing the number of structural parameters, and thus the training speed is greatly improved and the problem of overfitting is not easily caused [28]. Therefore, GRU is selected to construct a deep GRU neural network for behavioral depth feature extraction. The structure of a GRU recurrent network is shown in Figure 5. The GRU block is composed of an update gate and reset gate. How the GRU works is described below.

The update gate is used to control the degree to which the state information of the previous moment is retained in the current state. The higher the value of the update gate is, the more the state information of the previous moment is retained. The update gate state $z_{t}$ of time *t* is completed by the following formula:

(10) $$z_{t}=\sigma \left(W_{z}\cdot \left[h_{t-1},x_{t}\right]+b_{z}\right).$$

In the reset gate, the GRU calculates the reset gate state $r_{t}$ at a given time *t* to indicate how much past information needs to be forgotten, and the smaller the reset gate value is, the more information is ignored. The gate performs the following equation:

(11) $$r_{t}=\sigma \left(W_{r}\cdot \left[h_{t-1},x_{t}\right]+b_{r}\right).$$

The state ${\tilde{h}}_{t}$ of the GRU memory unit at the current moment is calculated as follows:

(12) $${\tilde{h}}_{t}=tanh\left(W_{h}\cdot \left[r_{t}\cdot h_{t-1},x_{t}\right]+b_{c}\right).$$

The output state $h_{t}$ of the GRU at the current moment is determined by

(13) $$h_{t}=\left(1-z_{t}\right)\cdot h_{t-1}+z_{t}\cdot {\tilde{h}}_{t}.$$

where, $x_{t}$ is the input vector for the *t*th time step. $h_{t-1}$ represents the output state of the GRU from the previous time step. $W_{z}$, $W_{r}$ and $W_{h}$ are the weight matrix of the hidden layer neurons of the update gate, reset gate and memory unit, respectively; $b_{z}$, $b_{r}$ and $b_{c}$ are the threshold vectors of the hidden layer neurons of the update gate, reset gate and memory unit, respectively, and they are all training parameters. The operator “ · ” represents point multiplication operation. σ and tanh represent sigmoid and hyperbolic tangent activation functions, respectively, and their calculation formulas are as follows:

(14) $$\sigma \left(x\right)=\frac{1}{1+e^{-x}}.$$

(15) $$\tanh\left(x\right)=\frac{e^{x}-e^{-x}}{e^{x}+e^{-x}}.$$

The labeled training data set can be expressed as $S_{2}=\left\{\left(R_{1},L_{1}\right),...,\left(R_{s},L_{s}\right),...,\left(R_{S},L_{S}\right)\right\}$, where *S* is the number of samples of similar activities, and $R_{s}$ is the RSSI measurement matrix of the *s*th window, $L_{s}$ is the label of the corresponding segment. For sample $R_{s}={\left[r_{s,0},...,r_{s,w},...,r_{s,W}\right]}^{T},\left(1\leq s\leq S\right)$, where $r_{s,w}\left(1\leq w\leq W\right)$ is the fusion vector of RSSI measurements of each link at time *w*, and the dimension is 1×J. $r_{s,w}$ is the input vector $x_{t}$ for GRU at time *w*. The GRU time step is equal to the window size *W*.

### 4.3. The Hierarchical Recognition Method

Compared with the deep learning method, the model constructed by using the traditional pattern-based method has lower complexity and lower requirements in terms of computing equipment. Therefore, the SVM model was constructed based on the traditional pattern method, which can achieve high accuracy for most activities in the coarse-level recognition stage. In order to solve the problem of the SVM model misidentifying some similar activities, the recognition model of similar activities based on the GRU model is constructed by the deep learning method in the fine-level stage. The deep learning method was only used to build the similar activity recognition model, rather than of all the activities, so the benefits of this operation were not only to build a lightweight deep learning network without needing to build a complex deep learning model, but it can also achieve a better recognition accuracy, also effectively reducing the training costs of the deep learning model. Assuming the set of activities that are difficult to recognize by the coarse-level recognition model is $L_{similar}\subset L_{label}$, and for a given sample $R_{s}$, the vector of the sample after statistical feature extraction is $f_{s}$, then the prediction label $F_{H}\left(R_{s}\right)$ of the hierarchical classification framework is represented by the following process:

(16) $$F_{H}\left(R_{s}\right)=\left\{\begin{array}{c} F_{2}\left(R_{s}\right),\;\;if\;F_{1}\left(f_{s}\right)\in L_{similar}\\ F_{1}\left(f_{s}\right),\;\;oetherwise \end{array}\right.$$

Among them, $F_{1}\left(\cdot \right)$ and $F_{2}\left(\cdot \right)$ represent the recognition functions of teh coarse-level and fine-level model, respectively.

## 5. Experimental Evaluation

### 5.1. Experimental Setup and Data Description

**Figure 6:**
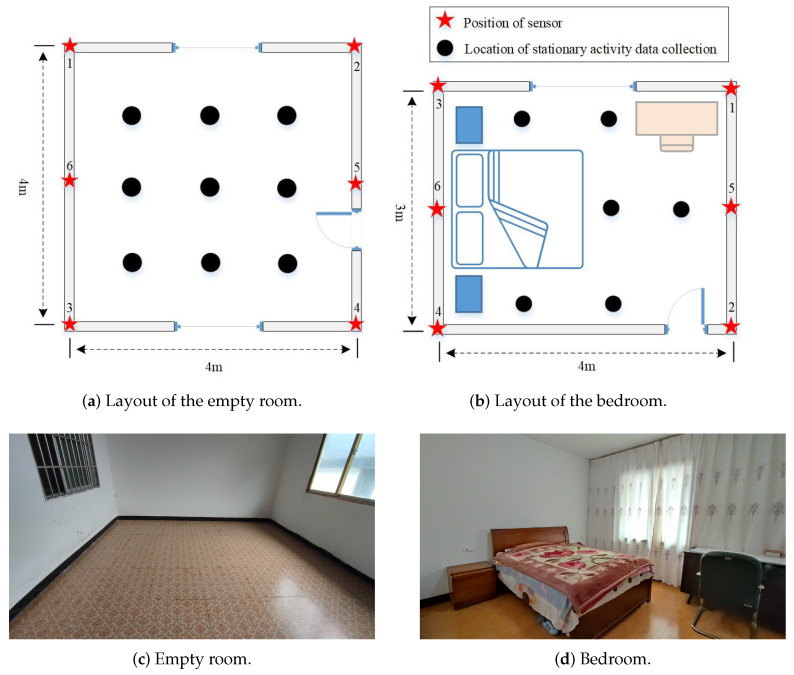
Layouts and photographs of two different indoor environments.

In Figure 6, we established two sensing networks to evaluate the performance of the proposed scheme in two typical scenarios, i.e., an empty room (4 m × 4 m × 3 m) and a bedroom (3 m × 4 m × 3 m). The sensor network consists of six sensor nodes which are equipped with ESP8266-12F chipsets and placed at six different positions (as shown by the red stars in Figure 6) 1.5 m above the ground. The sample frequency of the network is about 15 Hz, which is sufficient for estimating most activities. As shown in Figure 6, nodes 2–6 are stations (STAs), and node 1 consists of two ESP8266-12F chips, one for the station node and the other for the probe node to detect RSSI between it and each station node. Station node 1 transmits the RSSI measurement collected by probe node 1 to PC through the TCP protocol. All the nodes use the transmission power of 20 dBm and transmit over the frequency of 2.4 GHz. The hardware used for training the GRU model and signal classification is a computer equipped with Intel (R) Core (TM) i7-8550U CPU operating at 1.80 GHz with 8.0 GB of RAMa NVIDIA GeForce GTX 1080 Ti GPU.

As shown by the nine black circles in Figure 6a, there are nine feasible positions for the test object to collect three kinds of stationary activity signals, respectively, in the empty room, namely standing; sitting; and lying (lying on the ground). As shown by the six black circles in Figure 6b, there are six feasible positions for the test object to collect three kinds of static activity data, respectively, in the bedroom, namely standing; sitting; and lying. A sleeping activity was added in the second environment, where the volunteer was sleeping in two different positions in the bed, and the RSSI signal corresponding to this activity was collected at two different positions. In addition, the volunteer was asked to perform walking and running in two environments. The two activities had no fixed path but were spread over the entire viable area of activity. Finally, the RSSI signals were collected in both environments to enrich the categories of recognition in the case of the room being empty (i.e., no one exists in the environment). The data collected during two different time periods were used to form the training set and testing set, respectively. Taking the sliding window size 30 as an example, in the empty room, the samples used for the training set and testing set were 27,972 and 7019, respectively. In the bedroom, they were 33,120 and 8300, respectively.

Four measures were selected to evaluate the reliability of the model, namely accuracy; recall; precision; and f-score.

### 5.2. Results

In this section, the performance of the activity recognition was evaluated in two different experimental environments, namely an empty room and a bedroom. A sliding window of size 30 was used to cut the sensor signals, and a low-pass butterworth filter with a cutoff frequency of 5.25 Hz was used to filter out signal fluctuations. Then, an SVM classifier was constructed to realize the coarse recognition of activities. For activities with low recognition accuracies in the result of coarse recognition, a lightweight deep learning model was constructed by using the GRU algorithm to realize the fine recognition of similar activities. The activity recognition model was constructed by combining coarse-level and fine-level recognition models based on the proposed hierarchical classification method, and the recognition performance of the proposed method was evaluated according to four evaluation measures, namely the average accuracy; recall; precision; and f-score.

#### 5.2.1. Coarse-Level Activity Recognition Results

**Figure 7:**
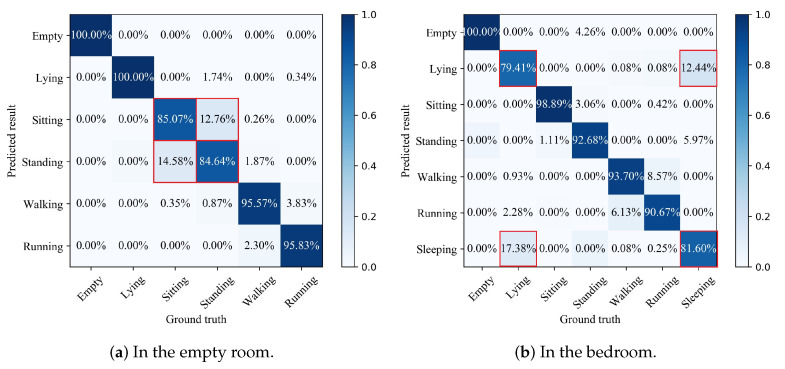
Confusion matrix of coarse-level recognition in the two environments.

The SVM method was used to construct a classfier for the statistical feature set, i.e., {Mean, SD, RMS, Max, Min, Range, MC, AM, ASD, AS, AK, SM} extracted from the sensing signals, to realize THE coarse recognition of activities. The kernel function of SVM adopted “polynomial” and its degree was selected to be 2. Due to the difference of environments, the SVM classifier constructed in the two scenarios presented different recognition results. Figure 7 shows the results of coarse-level activity recognition in the two environments. As can be seen from Figure 7a, the constructed SVM classifier can achieve more than 95% recognition accuracy for four activities in the empty room, i.e., empty; lying; walking; and running. Sitting and standing have slightly lower accuracy. As can be seen from Figure 7b, the constructed SVM classifier can achieve more than 90% recognition accuracy for five activities in the bedroom, namely empty; sitting; standing; walking; and running, while the recognition accuracy of lying and sleeping was slightly lower. Although the coarse recognition model can achieve high accuracies for most activities, it has low recognition accuracies for some similar activities. For example, in the empty room, the proportion that misidentified sitting as standing was up to 14.58%, and the proportion that misidentified standing as sitting was 12.76%. In the bedroom, the proportion that misidentified lying as sleeping was up to 17.38%, and the proportion that misidentified sleeping as lying was 12.44%. It can be found by experimental results that all of these low accuracy activities are due to the difficulty in distinguishing between one from the other.

#### 5.2.2. Results Based on Hierarchical Recognition Model

**Figure 8:**
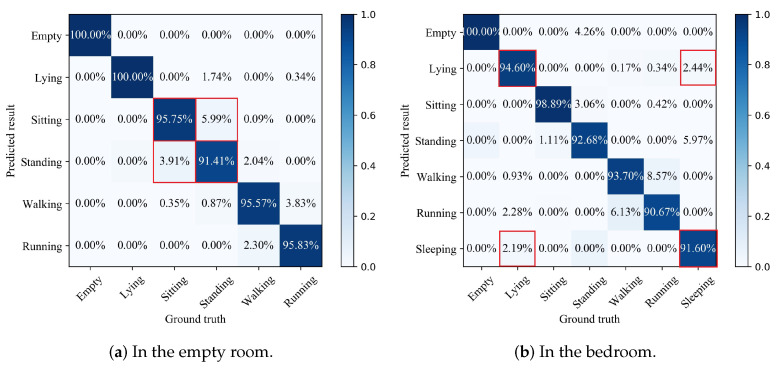
Confusion matrix of the hierarchical recognition in the two environments.

Designed for similar activities that are difficult to distinguish in coarse-level recognition results, namely sitting and standing in an empty room, and lying and sleeping in the bedroom, respectively, we used the GRU algorithm to build a lightweight deep learning model for such activities in the stage of fine recognition. By using the constructed deep GRU model to automatically extract feature description from the time-dependent relationship of the sensor signal, the complex nonlinear relationship between the input signal and the output tag can be effectively captured, and the activity can be recognized with high precision without the manual design features. Finally, the coarse-level and fine-level recognition models were combined into a hierarchical recognition model for activity recognition. Figure 8 shows the details of the recognition results in two experimental environments.

Figure 8a shows the probabilities of the true class versus predicted class, translating into the classification accuracy of 100%; 100%; 95.75%; 91.41%; 95.57%; and 95.83% for empty; lying; sitting; standing; walking; and running, respectively. Figure 8b shows the probabilities of the true class versus predicted class translating into the classification accuracy of 100%; 94.6%; 98.89%; 92.68%; 93.7%; 90.67%; and 91.6% for empty; lying; sitting; standing; walking; running; and sleeping, respectively. We used the results of the coarse recognition as baselines to demonstrate the performance of our proposed algorithm. Compared with the results of the coarse activity recognition, the accuracy of the proposed method for sitting and standing in the bedroom increased from 85.07% to 95.75% and from 84.64% to 91.41%, respectively, and the accuracy of sitting and standing in the bedroom increased from 79.41% to 94.6% and from 81.6% to 91.6%, respectively. The experimental results show that the proposed hierarchical recognition model can achieve higher accuracy for all activities, and the recognition accuracy of similar activities can be effectively improved.

### 5.3. Impact of the Hidden Units Number

**Figure 9:**
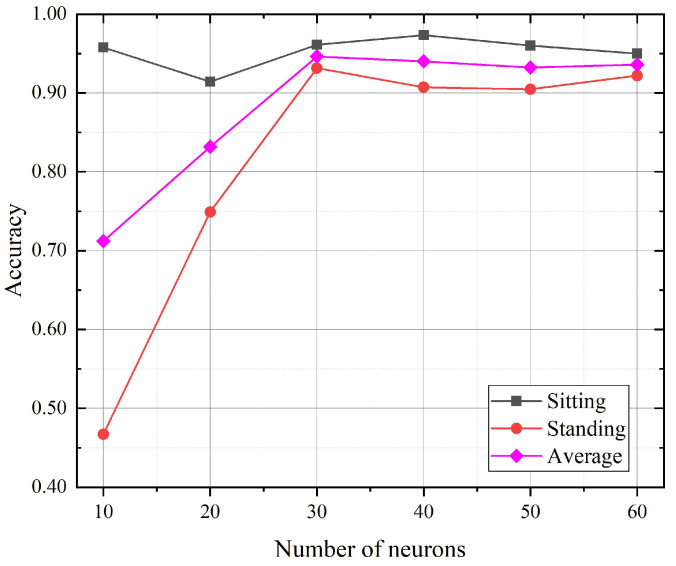
The accuracies for activity recognition with a different hidden units number.

We tried to explore the impact of the hidden units number of the GRU model on the fine-level activity recognition accuracy. Figure 9 shows the activity fine recognition results under teh different number of hidden units in the empty room. According to the experimental results, with the increase in the hidden units number, the average recognition accuracy increased slightly, and then gradually tended towards being stable. When the number of hidden units was set below 30, the average recognition accuracy significantly increased with the number of hidden units. Whereas when the number of hidden units was set between 30 and 60, the recognition average accuracy was stable around 94%. We also found that the recognition accuracy for standing will be poor with too few numbers of the hidden units, i.e., 10 and 20. Taking the computational consumption and the performance into consideration, we chose 30 as the number of hidden nodes.

### 5.4. Impact of the Sliding Window Size

**Figure 10:**
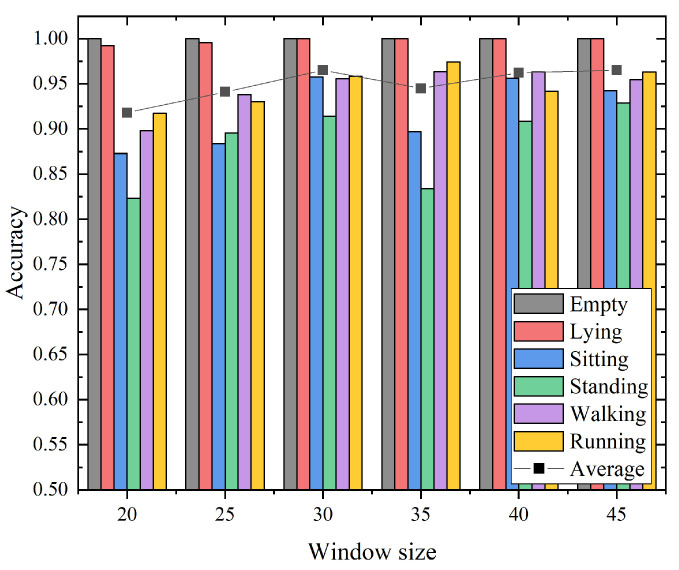
The accuracies for activity recognition with different window size.

In this section, we conducted experiments in the empty room to investigate the impact of the sliding window size on the hierarchical recognition of activities. The window size was set from 20 to 45 with the step size of 5. The recognition accuracy with different window sizes for our proposed approach is shown in Figure 10. According to the experimental results, with the increase in window size, the average recognition accuracy increases slightly, and then gradually tends to be stable. The overall accuracy fluctuates with the sliding window size within 5%. Concretely, when the window size increases to 30, the average recognition accuracy reaches the maximum value, i.e. 96%. After that, as the window size increases, the average recognition accuracy shows no obvious trend of growth. It is worth noting that when the window size was 30, the recognition accuracy for all activities exceeded 90%. Considering that a larger window size will increase the computational complexity, the system latency increases, which is undesirable for a real system. Hence, in order to guarantee the performance of the proposed system, the window size should be carefully selected. Experimental results show that the sliding window size of the proposed method is 30, which is the best trade-off.

### 5.5. Impact of the Number of Sensing Nodes

**Figure 11:**
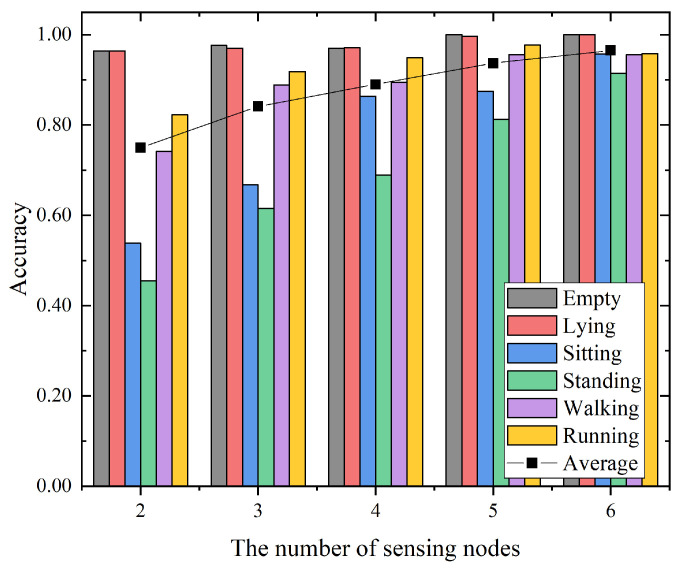
The accuracies for activity recognition with different sensing nodes.

To investigate the impact of the number of sensing nodes for the proposed hierarchical recognition model, we performed an additional experiment with the data from the different number of sensing nodes in the empty room. We started with the data from all the sensing nodes, i.e., six sensing nodes. Then, we randomly discarded the data from one sensing node at a time. Figure 11 presents the experimental results with the number of sensing nodes from 2 to 6 for the proposed hierarchical recognition model. The overall trend indicates that the average accuracy increases with the increase in the number of sensing nodes. Meanwhile, the recognition accuracy of sitting, standing, walking and running increases significantly, whereas the accuracy of empty and lying changes slightly. When the number of sensing nodes increased from 4 to 5, the average accuracy exceeded 90%, which was 93.7%. When the number of sensing nodes was 6, the average recognition accuracy reached 96.5%. The experimental results show that with the increase in the number of sensing nodes, the sensing information provided for the recognition model was more abundant, and the accuracy of recognition was also improved accordingly. Based on this test, we can roughly determine the importance of the number of sensing nodes.

### 5.6. Impact of the Statistical Features

In this section, the time domain statistical feature set {Mean, SD, RMS, Max, Min, Range, MC}, the frequency domain statistical feature set {AM, ASD, AS, AK, SM} and the combination of the time domain and the frequency domain feature set {Mean, SD, RMS, Max, Min, Range, MC, AM, ASD, AS, AK, SM} are, respectively, used to characterize activity-sensing signals, to study the influence of statistical features on the performance of the proposed hierarchical recognition model. Table 2 shows the results under a different set of features in the empty room. As can be seen from Table 2, the average recognition accuracy was 74.28% when only the frequency domain feature set was used for activity signal characterization. The combination of feature sets in the frequency domain and the time domain of sensing signals was used to represent human activity, with the highest average recognition accuracy of 96.50%. However, compared with the time domain feature set alone, the average recognition accuracy was improved by 0.77%, which was not too obvious. The above results indicated that the combination of time domain feature set and frequency domain feature set could achieve a better recognition effect than the single feature set, but the time domain feature set played the most prominent role in the activity representation.

### 5.7. The Comparison of HAR Models

To further validate the proposed hierarchical recognition approach, comparisons were made with eight other classifiers, including logistic regression (LR), KNN, naive Bayes, decision tree (DT), multi-layer perceptron (MLP), SVM, CNN and GRU. In this experiment, we used the same initial training data set for all the approaches and explored their performances on the same testing data set.

Table 3 compares the overall performance of all methods according to the the precision, recall, and f-score. Overall, we found that the traditional pattern-based approaches, i.e., LR, KNN, naive Bayes, and DT had limited performance, due to the limited modeling ability of these approaches with regard to the complicated RSSI values. The deep learning method based on GRU extracts activity features from the time dependence of RSSI measurements, which can achieve a better performance than the traditional pattern-based approaches and CNN. However, the proposed coarse-to-fine hierarchical method, which combines SVM based on the traditional pattern approach and GRU based on the deep learning approach, can better capture the differences in activities, thus achieving a better overall performance than either.

**Figure 12:**
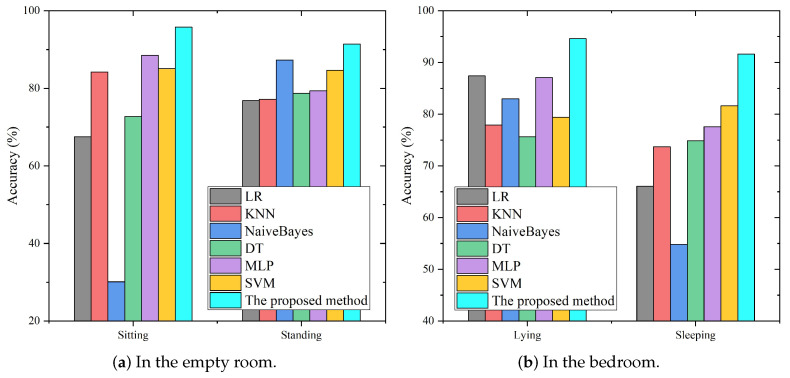
Comparison of the different methods for similar activity recognition.

Figure 12 compares the recognition results of all methods for similar activities in the empty room and the bedroom, respectively. For the example of the recognition results for sitting, the proposed hierarchical classification approach can achieve 95.75% accuracy, followed by MLP, SVM, KNN, DT and LR, which acheived 88.54%, 85.07%, 84.20%, 72.74% and 67.53%, respectively. Naive Bayes showed the worst performance, with the recognition accuracy rate of sitting only 30.12%, and the proportion of misrecognizing sitting as standing was as high as 63.37%. Compared with other traditional pattern-based methods, the proposed method achieved more than 90% accuracy for all activities and effectively solved the problem of low recognition accuracy for similar activities.

**Figure 13:**
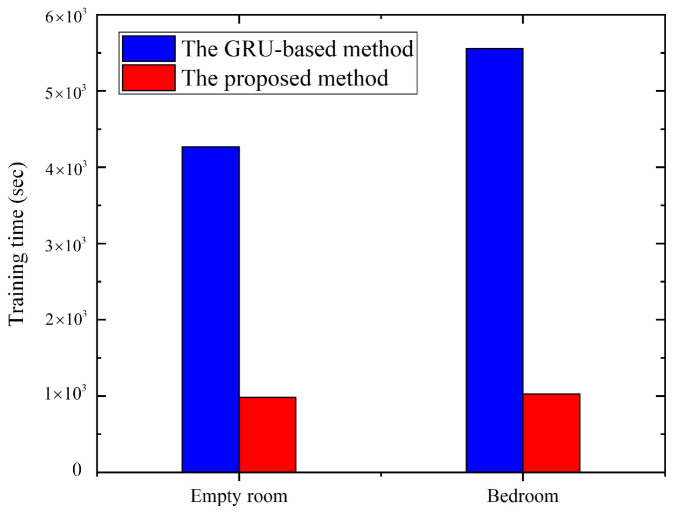
The comparison of the training times between two different methods.

Figure 13 shows the comparison of the training time between the proposed method and the method directly trained by GRU for all activities. From the perspective of training time, such as in the bedroom, the time required by the deep learning method based on GRU to build the model for all activities is at most 5558.34 seconds. The training time of the proposed method is 1028.43 seconds, including 819.83 seconds for the fine-level deep learning model construction, which is one-fifth of the deep learning method based on GRU.

To summarize the in-depth investigation of the activity recognition method, the proposed hierarchical recognition method provides an effective scheme for activity recognition based on WiFi-RSSI to improve performance and accelerate model construction.

## 6. Conclusions

In this paper, we proposed an RSSI-based passive HAR method based on a coarse-to-fine hierarchical deep learning framework. The ESP8266 sensor was applied to acquire RSSI data, which can greatly reduce deployment costs. In the proposed HAR framework, the SVM classifier based on the pattern method was constructed for coarse-level activity recognition and the GRU neural network was trained to realize the fine-level recognition of similar activities. The experimental results show that the proposed hierarchical classification framework can quickly and accurately identify the different and similar human activities. The overall recognition accuracy in the two experimental environments, i.e. the empty room and bedroom, were 96.45% and 94.59%, respectively. The proposed method yielded better performance compared with other existing methods. In addition, the training time of the proposed hierarchical model can be reduced by five times that of the standard deep learning model. In our future work, we will attempt to explore the hierarchical model considering the challenging factor of the environmental applicability for HAR in the context of smart homes.

## Figures and Tables

**Table 1:** Frequency domain statistical features.

Statistic	Expression
Amplitude Mean	$\mu_{amp}=\frac{1}{W}\sum_{i=1}^{W}C(i)$
Amplitude Standard Deviation	$\delta_{amp}=\sqrt{\frac{1}{W}\sum_{i=1}^{W}[C(i)-\mu_{amp}{]}^{2}}$
Amplitude Skewness	$\frac{1}{W}\sum_{i=1}^{W}[\frac{C\left(i\right)-\mu_{amp}}{\delta_{amp}}{]}^{3}$
Amplitude Kurtosis	$\frac{1}{W}\sum_{i=1}^{W}[\frac{C\left(i\right)-\mu_{amp}}{\delta_{amp}}{]}^{4}-3$
Shape Mean	$\mu_{shape}=\frac{\sum_{i=1}^{W}i\cdot C(i)}{\sum_{i=1}^{W}C(i)}$

**Table 2:** Performance comparison of the different feature sets for activity recognition in the empty room.

Statistical Feature Set	Precision	Recall	F-Score
	(%)	(%)	(%)
Time Domain	95.75	95.73	95.73
Frequency Domain	75.07	74.28	74.39
The Combination Set	96.49	96.45	96.46

**Table 3:** Performance comparison of the different methods for activity recognition in the empty room.

Method	Precision	Recall	F-Score
	(%)	(%)	(%)
LR	86.58	86.31	86.36
KNN	90.82	90.54	90.63
NaiveBayes	87.48	81.25	82.42
DT	88.89	88.00	88.26
MLP	93.01	92.69	92.77
SVM	93.62	93.59	93.60
CNN	88.91	88.93	88.90
GRU	95.56	95.53	95.53
The Proposed Method	96.49	96.45	96.46

## Data Availability

Not applicable.
